# Comprehensive Management of Takayasu Arteritis Using Immunologic and Antithrombotic Interventions With Cerebral Circulation Support: A Case Report

**DOI:** 10.7759/cureus.45137

**Published:** 2023-09-12

**Authors:** Hideo Kihara, Takafumi Uchi, Shingo Konno, Sayaka Takenaka, Hideto Kameda

**Affiliations:** 1 Neurology, Toho University Ohashi Medical Center, Tokyo, JPN; 2 Rheumatology, Toho University Ohashi Medical Center, Tokyo, JPN

**Keywords:** bed rest, methotrexate, tocilizumab, transient ischemic attacks, ischemic stroke, takayasu arteritis

## Abstract

A 20-year-old woman with bilateral common carotid artery stenosis was diagnosed with Takayasu arteritis (TA). She suffered from a recurrent stroke, and repeated transient ischemic attacks (TIAs) occurred due to middle cerebral artery (MCA) stenosis. The clinical course indicated that TA contributed to MCA stenosis despite the negative results for serological inflammation markers. Immunotherapy with tocilizumab and methotrexate effectively reduced disease activity and improved symptoms. Bed rest and hydration prevented further TIAs and ischemic stroke progression. Long-term monitoring of neurological symptoms and angiography findings is essential to evaluate disease severity and treatment efficacy in TA patients with negative inflammatory markers.

## Introduction

Takayasu arteritis (TA) is a chronic inflammatory vasculitis affecting large vessels, particularly the aorta and its main branches. It is characterized by vessel inflammation that leads to wall thickening, fibrosis, and stenosis. The patient’s condition is idiopathic and granulomatous [1]. Although the condition predominantly affects large vessels such as the aorta and its main branches, it can also involve extracranial and intracranial vessels [2,3]. Inflammation and intimal proliferation lead to wall thickening, stenotic or occlusive lesions, and thrombosis, while arterial wall destruction can cause aneurysms and dissections. As the disease progresses, it can cause cerebral ischemia, stroke, and other neurological complications [4].

A case of a 20-year-old woman with cerebral infarction caused by TA, who was treated conservatively and discharged without disabilities, is reported.

## Case presentation

**Figure 1:**
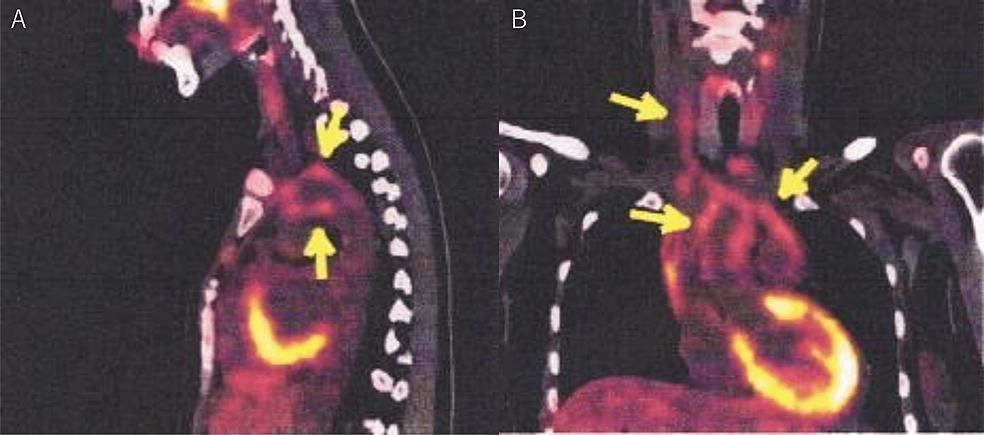
18F-fluorodeoxyglucose positron emission tomography. There was an accumulation of 18F-fluorodeoxyglucose in the vessel wall from the ascending aorta to the aortic arch (SUVmax 2.93-3.06) (yellow arrows on the aorta in A and B) and from the brachiocephalic artery to the right common carotid artery (SUVmax 2.53-2.56) (yellow arrow in the cervical area in B). SUV: standardized uptake value.

The patient experienced frequent transient defects in only the right visual field and weakness in the right upper limb. Cardiovascular computed tomography (CT) angiography performed six months after the initial manifestations revealed occlusion of the right common carotid artery (CCA) and severe stenosis of the left internal carotid artery (ICA) and bilateral subclavian arteries. Carotid echocardiography showed bilateral macaroni signs in the CCAs. Serologically, negative myeloperoxidase anti-neutrophil cytoplasmic antibody (ANCA) and proteinase 3-ANCA, antinuclear antibodies (<1:40), and normal levels of IgE and eosinophils. 18F-fluorodeoxyglucose (FDG) positron emission tomography scan revealed FDG accumulation in the vessel wall from the ascending aorta to the aortic arch and from the one isthmus artery to the right and left CCA (Figure 1).

**Figure 2:**
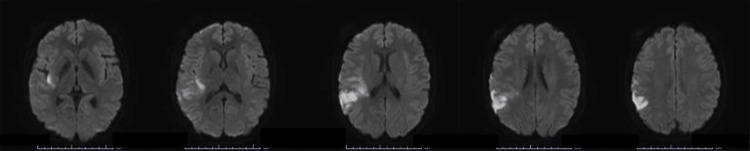
Brain MRI performed on day 0. Axial diffusion magnetic resonance imaging showed high-intensity signals in the right frontal, temporal, and parietal lobes.

Based on the above findings, TA was diagnosed based on the criteria defined by the Japan Circulation Society 2017 Guidelines on Managing Vasculitis Syndrome [5]. Treatment with oral prednisolone (PSL) (50 mg/day), equivalent to 1 mg/kg/day, and tocilizumab (162 mg/week) was initiated, along with aspirin (100 mg/day), considering the risk of cerebral infarction. Thirty days after the initiation of immunotherapy, the patient visited our department because of the sudden onset of left-sided weakness and sensory numbness (day 0). At this time, she continued to receive PSL (30 mg/day), tocilizumab at the same dose as the initiation, and aspirin (100 mg/day). Magnetic resonance imaging (MRI) revealed an acute cerebral infarction in the right temporal lobe (Figure 2).

**Figure 3:**
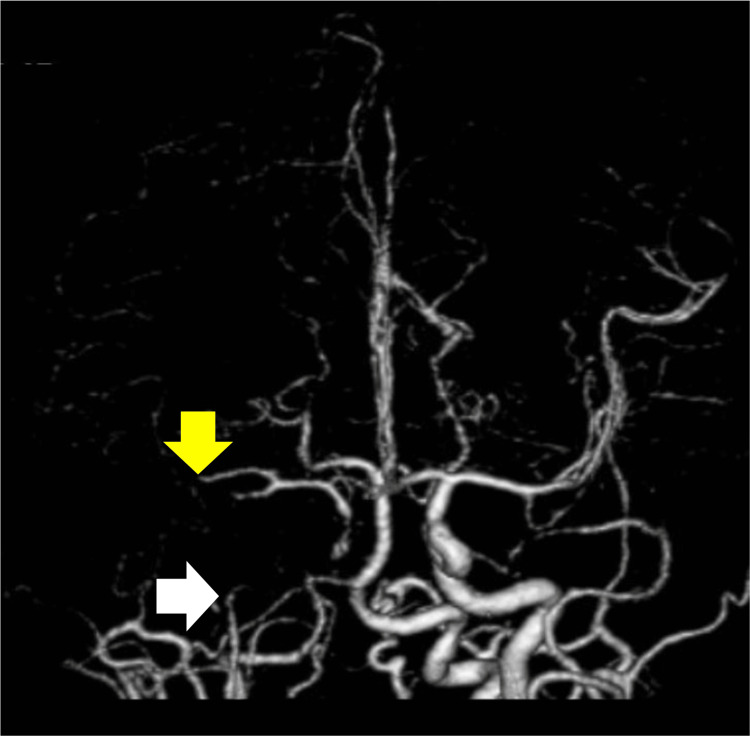
Brain three-dimensional CT angiography on day 0 (frontal view). On day 0, a three-dimensional CT angiogram showed a stenosis-like appearance in the right intracranial internal carotid artery (white arrow) and middle cerebral artery (yellow arrow).

Brain three-dimensional CT angiography (3D-CTA) showed disturbed blood flow in the intracranial ICA and a branch of the M2 portion of the right middle cerebral artery (MCA) (Figure 3).

The patient’s vital signs were stable, but the physical examination revealed mild weakness and hyperactive reflexes in the left upper and lower limbs. However, the results of blood tests related to inflammation were all negative: WBCs, 7700/µL; CRP, <0.01 mg/dL; erythrocyte sedimentation rate (ESR), 3 mm/60 min; and D-dimer, <0.5 mg/dL (some findings were positive pre-immunotherapy: CRP, 0.88 mg/dL and ESR, 47 mm/60 min). Factors related to atherosclerosis, cholesterol, glucose, and uric acid were normal. The patient was treated for acute ischemic stroke caused by main cerebral artery stenosis with argatroban (600-200 mg/day) on days 0-7 and edaravone (60 mg/day) on days 0-14, following which clopidogrel (75 mg/day) was added to immunotherapy.

**Figure 4:**
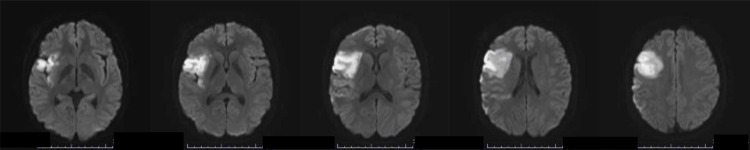
Brain MRI performed on day 51. Axial diffusion magnetic resonance imaging showed new high-intensity signals in the right frontal lobe.

Repeated transient weakness developed in the left upper and lower extremities despite starting anticoagulation therapy. To protect the vascular endothelium, atorvastatin (5 mg/day), ethyl omega-3 fatty acid (2 g/day), and ibudilast (10 mg/day) were added to the treatment regimen. Furthermore, on days 10-15, transient ischemic attacks (TIAs) like episodes developed frequently when the patient stood and walked. MRI revealed a new infarction in the right frontal lobe (Figure 4).

**Figure 5:**
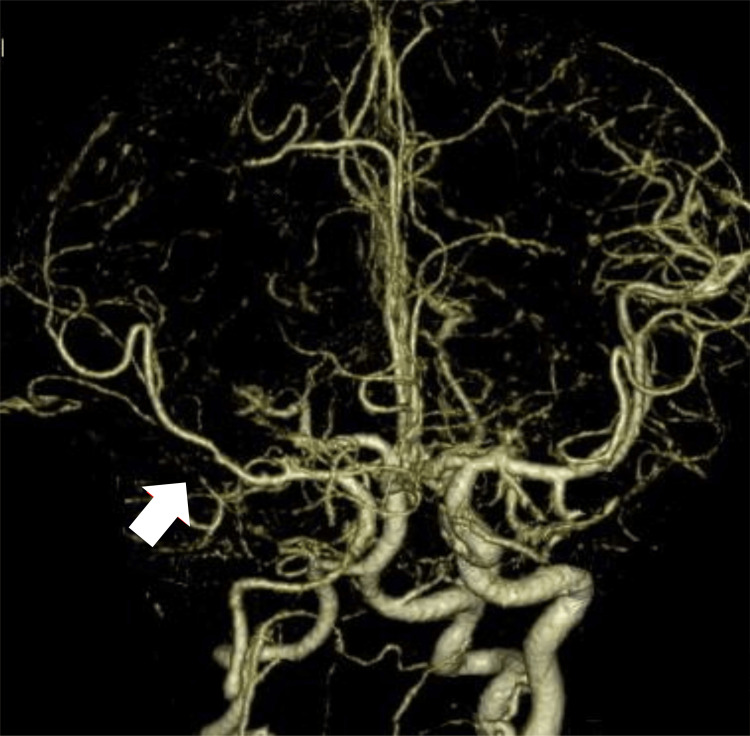
Brain three-dimensional CT angiography on day 52 (frontal view). On day 52, brain CT angiography showed improving blood flow in the peripheral segment from the M1 portion to the middle cerebral artery, with remaining stenosis over the entire length of the vessel.

Although the patient received a moderate dose of PSL, cholesterol, glucose, and uric acid remain normal. Considering the possibility of TA-induced stenosis progression in the MCA, methotrexate (8 mg/week) was started and gradually increased to 16 mg/week. To dilate the intracranial artery, cilostazol (200 mg/day) was also administered. The systolic blood pressure in the standing and supine positions showed a 20-mmHg difference, and lying in bed with continuous infusion of sufficient extracellular fluid resolved the TIAs. After treatment with two immunosuppressive agents in addition to intensified antithrombotic therapy, MRI on day 52 revealed no enlargement of the infarcted foci. Compared to 3D-CTA on day 0 (Figure 3), that of on day 52 showed improved blood flow in the M1 to peripheral segments of the MCA (Figure 5).

**Figure 6:**
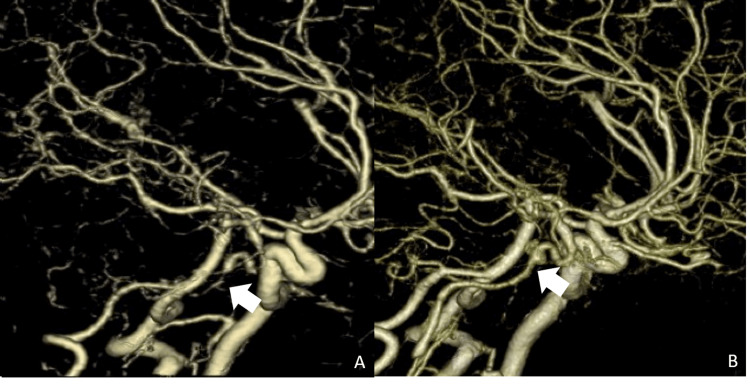
Brain tree-dimensional CT angiography performed on days 0 and 76 (lateral view). (A) On day 0, brain CT angiography showed stenosis of the intracranial internal cerebral artery (white arrow). (B) On day 76, brain CT angiography showed better blood flow in the right middle cerebral artery and the internal carotid artery (white arrow).

Compared to 3D-CTA on day 0 (Figure 6A), that of day 76 revealed slightly improved blood flow in the right ICA and MCA (Figure 6B). From the same day, the hydration and bed lying times were tapered. On day 79, the patient could walk freely, and the infusion was stopped. The patient experienced no recurring symptoms and was transferred to a rehabilitation hospital.

## Discussion

This patient developed an ischemic stroke and multiple TIAs due to bilateral stenosis of the CCAs and the right M2 segment of the MCA. TA is known to affect both right and left CCAs, while the stenosis of the M2 segment was a newly developed lesion. The immunotherapy provided by the previous hospital normalized the patient’s inflammatory marker levels.

One important consideration in such cases is identifying which etiology of stenosis in the M2 branch, TA or thrombophilia, was highly possible as an adverse effect of glucocorticoids. Glucocorticoid use is associated with cerebral venous thrombosis [6], which is closely related to ischemic stroke; however, the relationship is complex and not fully understood. TA can cause stenosis in the territory of high-grade stenosis of the MCA [7]. The best serological marker indicating TA activity is ESR, but normalization of ESR is not always an indicator of recovery in patients with TA [8]. Generally, patients with TA often show non-specific clinical symptoms and elevated levels of the serum markers of inflammation [9], but these markers have limited sensitivity and specificity [10]. Therefore, patients with TA treated with tocilizumab, which masks elevated levels of inflammatory markers, should be diagnosed using a multimodal approach.

Local inflammation of the blood vessels is often not reflected serologically by elevated levels of inflammatory markers. Moreover, antithrombotic therapy does not prevent the spread of ischemic strokes. Therefore, we believe that TA was still active in this patient and evaluated changes in neurological symptoms and angiography findings as indicators of disease severity and therapeutic effect.

The treatment options for TA include immunotherapy and surgery. The former consists of corticosteroids [11], tocilizumab [12], and methotrexate [13], while the latter consists of endovascular stenting and balloon dilatation. Because this patient was possibly in the active phase, surgical treatment was avoided. For example, a catheter operation from the aorta to the CCA lumen can induce embolism. Plastic bypass surgery from the right external carotid artery to the peripheral MCA was also not an option because of the possibility of restenosis.

Tocilizumab is effective in reducing disease activity and improving symptoms after three and six months of treatment, respectively, in patients with TA [14]. Another study found that tocilizumab was effective in treating refractory or severe TA after six months of treatment [12]. Tocilizumab combined with methotrexate was found to be more effective than tocilizumab alone [12]. Thus, the additional methotrexate administration may have contributed to the improved symptoms observed in the present case. Although the achievement of a stable TIA and ischemic stroke status requires long-term periods of up to 76 days after initiation of immunotherapy, the response to treatment varies among individuals. In this case, improved MCA stenosis demonstrated the importance of immunotherapy in aortitis from the early onset of symptoms to prevent scarring stenosis and occlusion of the vessel.

During the treatment course, the patient had frequent TIAs in the sitting and standing positions, which decreased intracranial blood flow. There is limited evidence regarding the effectiveness of bed rest in patients with unstable symptoms of cerebral infarction. Bed rest may be necessary for unstable symptoms of ischemic stroke caused by severe stenosis of the main intracranial artery or extracranial ICAs.

In this case, bed rest and adequate hydration prevented recurrent TIAs and stroke, like stroke patients with atherosclerotic intracranial and cervical vascular stenosis [15].

## Conclusions

This case of a 20-year-old woman with TA, who developed cerebral infarction, underlines the critical role of conservative treatment. The approach, focusing on immunotherapy instead of surgery, showed promising outcomes, leading to no sequela upon discharge. Her improved symptoms and alleviated vascular complications reinforce the value of combination immunotherapy with tocilizumab and methotrexate in managing TA. In this case, blood test data for inflammation due to immunosuppressive therapy may be false-negative, and test results may not reflect true inflammation. For this reason, it is important to use a combination of different approaches and tests, not just the usual means of testing, to evaluate disease activity in TA patients receiving immunosuppressive therapy. This patient's course of treatment further emphasizes the importance of a multifaceted diagnostic approach, careful symptom monitoring, and long-term management strategies in patients with TA.
